# 0984. Perfusate from lungs ventilated *ex-vivo* with high tidal volumen induce *in vitro* endotelial dysfunction reversed by superoxide dismutase and tempol

**DOI:** 10.1186/2197-425X-2-S1-P69

**Published:** 2014-09-26

**Authors:** L Martínez-Caro, I Ortiz, A Sanchez-Ferrer, Y Rojas, L Smit, B de Olaiz-Navarro, A Ferruelo, N Nin, A Esteban, JA Lorente

**Affiliations:** Centro de Investigación Biomédica en Red de Enfermedades Respiratorias (CIBERES), Getafe, Spain; Intensive Care Service and Burn Unit, Hospital Universitario de Getafe, Getafe, Spain; Hospital Universitario de Getafe, Getafe, Spain; Pediatric Intensive Care Service, Hospital Virgen de la Salud, Toledo, Spain; Universidad Alfonso X, Madrid, Spain; Intensive Care Service, Hospital de Torrejon, Madrid, Spain; Intensive Care Service, Hospital Español, Montevideo, Uruguay

## Introduction

Ventilator-induced lung injury (VILI) has been related not only to pulmonary injury but also to systemic damage. We performed a bioassay using ex vivo models of VILI and of vascular function in order to determine the role of pulmonary-derived factors in ventilator-induced endothelial dysfunction. The involvement of nitro-oxidative stress was also examined [1].

## Objectives

(i) To demonstrate that the release of soluble factors derived from the lung induces vascular endothelial dysfunction.
(ii) To define the role of nitro-oxidative stress in ventilator-induced endothelial dysfunction.

## Methods

Ex vivo ventilated and perfused lungs (Harvard Apparatus, MA) from male Sprague-Dawley rats (weight 325-375 grams) were subjected to high tidal volume (V_T_=25 mL/kg + PEEP=0 cm H_2_O) mechanical ventilation for 2.5 h (n=22). Lungs were perfused (4 mL/min) with Krebs solution + 4% albumin (bubled with 5% CO_2_ and 20% O_2_) that was recirculated throughout the experiment. Aortic rings extracted from healthy rats were incubated in an organ bath for 60 minutes with the perfusate collected from the ventilated lungs. Endothelium-dependent relaxation was measured in norepinephrine precontracted rings (acetylcholine, 10 nM-10 uM). Superoxide dismutase (SOD 100 u/ml) or tempol

(10^-4^ M) (extracellular and intracellular superoxide scavengers, respectively) or MnTMPyP (10^-5^ M) (a superoxide and peroxynitrite scavenger), were added to the organ bath in order to explore the role of nitro-oxidative stress in vascular dysfunction. Dose-response curves were compared by repeated-measurements ANOVA. We followed the Principles of Laboratory Animal Care (2010/63/UE 22-09, RD 53/2013 BOE 1-02, ley 32/2007 BOE 7-11).

## Results

High V_T_ mechanical ventilation was associated with an increase in peak airway pressure (PIP), as well as increased levels of LDH, CK and lactate in the perfusate at the end of the experiment, in approximately half of the high V_T_ ventilated lungs (n=10), whereas half of the isolated-perfused lungs did not show any changes in PIP, LDH, CK and lactate after 2.5 h of high V_T_ mechanical ventilation (n=12). The perfusate collected from the lungs that showed increased PIP induced an impairment in vascular responses in vitro. On the contrary, the perfusate collected from lungs that did not show an increase in PIP did not induce significant changes in vascular responses in aortic rings. Impaired-responses to acetylcholine were improved by the administration of tempol and SOD, but not by MnTMPyP, to the organ bath (n=12-15 rings per treatment).

## Conclusions

(i) Factors released from injured lungs ex vivo are able to induce endothelial dysfunction in vitro.
(ii) Oxidative stress is involved in endothelial dysfunction induced by high V_T_ mechanical ventilation.
